# Genetic Resources of Cereal Crops for Aphid Resistance

**DOI:** 10.3390/plants11111490

**Published:** 2022-05-31

**Authors:** Evgeny E. Radchenko, Renat A. Abdullaev, Irina N. Anisimova

**Affiliations:** N. I. Vavilov All-Russian Institute of Plant Genetic Resources, 42-44 Bolshaya Morskaya Street, St. Petersburg 190000, Russia; abdullaev.1988@list.ru (R.A.A.); irina_anisimova@inbox.ru (I.N.A.)

**Keywords:** plants, insects of the Aphididae family, phytophage–host plant interaction, resistance mechanisms, resistance genes

## Abstract

The genetic resources of cereal crops in terms of resistance to aphids are reviewed. Phytosanitary destabilization led to a significant increase in the harmfulness of this group of insects. The breeding of resistant plant genotypes is a radical, the cheapest, and environmentally safe way of pest control. The genetic homogeneity of crops hastens the adaptive microevolution of harmful organisms. Both major and minor aphid resistance genes of cereal plants interact with insects differentially. Therefore, rational breeding envisages the expansion of the genetic diversity of cultivated varieties. The possibilities of replenishing the stock of effective resistance genes by studying the collection of cultivated cereals, introgression, and creating mutant forms are considered. The interaction of insects with plants is subject to the gene-for-gene relationship. Plant resistance genes are characterized by close linkage and multiple allelism. The realizing plant genotype depends on the phytophage biotype. Information about the mechanisms of constitutional and induced plant resistance is discussed. Resistance genes differ in terms of stability of expression. The duration of the period when varieties remain resistant is not related either to its phenotypic manifestation or to the number of resistance genes. One explanation for the phenomenon of durable resistance is the association of the virulence mutation with pest viability.

## 1. Introduction

The phytosanitary destabilization caused by the biocenotic balance disruption, which entailed significant economic losses, and the expansion of the composition of dominant harmful organisms has become prolonged. In recent years, many regions of the world have witnessed an increase in the harmfulness of aphids that feed on cultivated plants. For instance, only in the western United States, an outbreak of the Russian wheat aphid *Diuraphis noxia* (Kurdjumov) has caused a loss of wheat and barley yield worth over one bln dollars within 20 years since 1986 (the first detection of the pest) [1]. Another invasive phytophage, the hedgehog grain aphid *Sipha maydis* Passerini, was first discovered in the USA in 2007 on *Leymus condensatus* (J. Presl) Á. Löve, and since then, has infested almost all cereal crops in vast areas [2,3]. Another recent example is the infestation of sorghum fields by the sugarcane aphid, *Melanaphis sacchari* Zehntner, since 2013. Since 2014, all sorghum fields in Louisiana and Mississippi have been infested with sugarcane aphids, and USD $10 million has been spent on protective measures. The yield losses of susceptible sorghum hybrids can reach as high as 60% [4].

The degree of cereal aphid harmfulness depends on the number of pests and the timing of their settling on plants, as well as on the duration of insects feeding [5,6]. The greatest damage to winter and spring crops is caused by the pests migrating to fields during the sprouting phase [7]. The harmfulness of aphids is also expressed in a decrease in the sowing and other consumer qualities of seeds [8,9]. The feeding of the greenbug *Schizaphis graminum* Rond. causes qualitative changes in the biochemical composition of plants and leads to serious changes in physiological processes: for example, free amino acids accumulate in the damaged leaves, which is usually observed in aging plant tissues. In resistant forms, the composition of metabolites changes in such a way that they become unappetizing or toxic, while the biochemical composition of susceptible plants becomes modified in such a way that the plants become attractive to insects for feeding [10]. As a result of the Russian wheat aphid reproduction on wheat plants, the content of chlorophyll decreases, and there occurs a selective inhibition of the synthesis and accumulation of proteins necessary for the normal life of plants [11,12]. At the same time, the increased concentration of free amino acids apparently promotes greater fertility of insects and the appearance of winged individuals [13,14]. In addition, the bright yellow color of infected plants attracts winged aphids [15]. Due to the rather weak ability of cereal aphids to distinguish between forage and non-forage plants, these pests can be important vectors of virus diseases in beets and potatoes [16,17].

According to the specific features of development and the composition of plants on which they feed, aphids are divided into two biological groups; that is, non-migratory and migratory. According to the place of feeding, the aphids can be distinguished as those that damage the above-ground organs of cereals or the roots. The most harmful and widespread are the aphids that feed on the above-ground organs of cereal plants.

The eggs of the non-migratory aphids usually winter on the leaves of winter cereal crops of perennial and wild grasses. In southern regions, overwintering of adults is possible. In spring, larvae hatch and develop into wingless females called fundatrix. The latter reproduce parthenogenetically and yield several generations. The emerging winged females fly to other plants, including spring grasses, where they continue reproducing. The number of aphid generations and fecundity depend mainly on weather conditions. After the spring crops have been harvested, insects feed on the sprouts from the shattered grain and wild-growing grasses and then migrate to the winter crop seedlings. In autumn, alate sexuparae appear, which give birth to larvae that turn into winged males and wingless females. After mating, females lay the eggs that winter [18]. Among the non-migratory species, the most harmful ones and most discussed in the literature are the greenbug *S. graminum*, the English grain aphid, *Sitobion* (*Macrosiphum*) *avenae* F., the Russian wheat aphid, *D. noxia*, and the corn leaf aphid, *Rhopalosiphum maidis* Fitch.

The migrating aphids live on grasses only in summer, and in the fall, they move to their primary host plants, usually trees or shrubs. Insects winter on them in the egg phase. In spring, larvae hatch from the eggs, giving rise to numerous colonies on the leaves. The deteriorating living conditions in trees force the aphids to change their lifestyle. The emerging winged females migrate to grasses, where the aphids reproduce parthenogenetically during the summer. In the fall, the winged individuals appear and migrate to the primary food plants. Among the migratory species, the most important are the bird cherry-oat aphid, *Rhopalosiphum padi* L., and the rose-grain aphid, *Metopolophium dirhodum* Walk.

One of the main reasons limiting the harmfulness of aphids on cereals is plant resistance. The breeding of resistant plant genotypes is a radical and, at the same time, the cheapest and most environmentally safe way to control aphids. The growth of losses makes the payback of breeding for resistance grow faster than its cost. Therefore, we overview the genetic diversity of major cereal crops (wheat, barley, oat, sorghum) in terms of aphid resistance and summarize the available information on the manifestation and genetic control of resistance traits. We also discuss the strategies for the rational use of genetic resources of cereal crops in breeding.

## 2. Types of Plant Resistance to Aphids

According to the generally accepted classification of R. Painter [19], which reflects the ecological aspects of the plant–arthropod interaction, three types (or, often, categories) of resistance are distinguished, namely nonpreference (antixenosis) [20]; that is, rejection of a plant when a choice is possible, antibiosis (adverse effect on the phytophage viability during feeding), and tolerance.

All types of resistance can be manifested simultaneously by one host plant and, moreover, can be determined by the same factor. For instance, cyclic hydroxamic acids and indole alkaloids contained in cereal plants can cause both antixenosis and antibiotic resistance to aphids [21,22]. Due to the absence of easily testable marker signs of antixenosis and antibiosis, the literature data on the relationships between resistance types are scanty and rather contradictory. Several genes of antixenosis, antibiosis, and tolerance were localized on five chromosomes in wheat-barley addition lines [23]. Different types of resistance of wheat and tritordeum (*Hordeum chilense* Roem. and Schult. × *Triticum turgidum* L., 2n = 42) to *S. graminum* and *D. noxia* were found to be controlled by different genes [24,25]. An analysis of resistance of 26 wheat accessions to the Russian wheat aphid and greenbug revealed a difference in the genetic systems that control the three types of insect resistance. Moreover, it was established that the development duration and aphid fecundity (i.e., manifestations of antibiosis) are also controlled by different genes [26]. Wheat accessions with the genes *Gb3*, *Gbx*, and *Gbz* are characterized by three types of resistance to the greenbug biotypes E and I; however, no antixenosis was detected during the interaction with biotype K [27]. On the other hand, the almost isogenic TXGBE273 line with the *Gb3* gene of resistance to *S. graminum* exhibits all three types of aphid resistance, which were not detected in a susceptible analog TXGBE281 [28]. In our experiments, the coincidence of segregation for antixenosis and antibiosis was found in cases of monogenic (*Sgr4* gene), digenic (*Sgr1*, *Sgr2, Sgr7*, and *Sgr8*) control of sorghum resistance to the greenbug, and the complementary interacting genes (*Sgr9*, *Sgr10*). In “Delphi 400” × “Siete Cerros 66” F_3_ wheat hybrids, the paired manifestation of antixenosis and antibiosis to bird cherry-oat aphid was also observed [29].

In our opinion, although different genetic control of antixenosis and antibiosis is possible, their identity is observed much more often. It is necessary to note two important consequences that follow from the common genetic control of two types of resistance:

(1) Both antibiosis and antixenosis equally cause the appearance of pest biotypes that overcome the resistance in the host plant;
(2) There is no need to breed resistant varieties separately for antibiosis and antixenosis since the weakly damaged genotypes selected from the segregating populations possess both types of resistance.

At the same time, it can be quite confidently asserted that the genetic nature of tolerance differs from that of antixenosis and antibiosis. Tolerance is usually associated with the high rate of development and the high compensatory response of plants, i.e., with pest nonspecific genetic systems.

## 3. Mechanisms of Passive (Constitutional) Resistance of Cereals to Aphids

According to N.I. Vavilov [30], the natural (inherent) resistance of plants to harmful organisms is subdivided into generic and specific (non-host) resistance (associated with the specialization of parasites and determined by the divergence of hosts and parasites in their evolution) and varietal resistance, which can be active (physiological, associated with an active reaction of host cells, accompanied by physiological and chemical reactions and neoplastic formations), structural (passive, mechanical, due to the morphological and anatomical characteristics of varieties), chemical (passive), or be determined by the damage evasion by plants due to early maturity. As N.I. Vavilov put it, resistance is the interaction of terms. The extensive literature discusses the mechanisms of passive and, more recently, active (induced) resistance of cereals to aphids.

The morphological and anatomical features of plants can ensure the resistance of cereal crops to aphids to a greater or lesser extent. Studies of the genetics of plant resistance to pests can be reduced to the study of the genetic control of certain morphological traits only in a number of cases. Undoubtedly, resistance is usually determined by several fundamentally different mechanisms, and it is possible to talk only about the analysis of morphological characters that are part of the total phenotypic variance.

The literature data on the relationship between pubescence and plant resistance to cereal aphids are somewhat contradictory. It has been shown that the density and length of trichomes are not the signs that mark wheat resistance to the greenbug and bird cherry-oat aphid [31,32]. At the same time, there are known cases of *R. padi* feeding on wheat varieties with dense leaf pubescence, when the insect number growth rate was much lower and aphids’ behavior more restless [33,34]. The leaves of PI 137739 accession, which is characterized by a pronounced antixenosis to *D. noxia*, have a long pubescence [35]. Antixenosis of synthetic hexaploids (*T. dicoccum* Schrank ex Shübl. × *Ae. tauschii*) to the Russian wheat aphid is also associated with leaf pubescence [36].

Barley plants without waxy coating were less colonized by *M. dirhodum* [37], while resistance to *R. padi* and waxy coating on leaves were closely correlated [38]. Japanese scientists made a field estimation of the aphid (mainly *R. maidis*) infestation of F_2_ plants from crosses of two *Hordeum vulgare* ssp. *spontaneum* K. Koch accessions characterized by green leaves (controlled by the *F9* gene) and waxy coating (*Gl3* gene), with an *H. vulgare* L. OUL 117 accession (yellow leaves without waxy coating, *gl3gl3 f9f9*). The alleles *Gl3* and *F9* ensured the highest level of resistance; the presence of either *gl3gl3* or *f9f9* homozygotes determined intermediate resistance, while the plants with yellow leaves without waxy coating were heavily infested by aphids [39]. X. Ni et al. studied the association of waxy coating with resistance to *D. noxia* [40]. The ultrastructure and chemical composition of the epicuticular wax on the leaves of resistant and susceptible wheat cultivars were similar but differed from the structure and composition of the wax on the leaves of the susceptible “Morex” barley cultivar and the resistant “Border” oat cultivar, which, in turn, differed, if at all, from each other. With the wax removed, “Morex” remained the most favorable, and “Border” the most resistant host.

Sorghum accessions, either without or with a slight waxy coating, are poorly infested by *S. graminum*. The bloomless is controlled by the recessive genes *bm1* and *bm2*; the sparse bloom is determined by the recessive genes *h1*, *h2,* and *h3*, while antixenosis does not manifest itself in the sprouting phase. “Shallu Grain” and IS 809 accessions inherited resistance regardless of the *bm* genes [41,42,43].

The literature on substances produced by plants for protection against phytophages is extremely extensive. The role of secondary plant metabolites, e.g., terpenoids, phenols, flavonoids, alkaloids, glucosinolates, etc., is widely discussed. An active protective role is played by protein compounds, primarily inhibitors of phytophage hydrolases (proteinases, β-amylases, etc.) and lectins. These substances are present mainly in the storage organs of plants, and the damage by insects induces their accumulation.

The leaves of aphid susceptible winter wheat varieties are characterized by an increased content of bound and free amino acids [44,45]. Aphid resistance is also associated with a high content of phenols and flavonoids in plant tissues [46,47]. The antibiotic resistance of winter wheat to the English grain aphid closely correlates with high values of the toxicity index, which reflects the ratio of free phenols to free amino acids content in a plant [48].

The toxic and antifidant effect of benzoxazinoids (BXDs—cyclic hydroxamic acids and their metabolites in cereal crop plants; i.e., DIMBOA, DIBOA, MBOA, etc.) on cereal aphids and other phytophages, as well as on pathogens, is discussed. For instance, various species of the Gramineae family with a relatively high concentration of BXDs are resistant to the greenbug [49]. It has been established by now that the synthesis of BXDs in maize is controlled by 16 genes (*ZmBx1*–*ZmBx14*, *ZmGlu1*–*ZmGlu2*) [50,51], by 13 in wheat (*TaBx1*–*TaBx5*, *TaGlu1a*–*TaGlu1d*, *TaGT1a*–*TaGT1d*) [52,53], and by eight *ScBx* genes in rye [54]. The plants of *Hordeum brachyantherum* Nevski, *H. flexuosum* (Nees ex Steud.) A. Love, *H. lechleri* (Steud.) Schenck, and *H. roshevitzii* Bowden were found to contain DIBOA. *H. lechleri* had the highest DIBOA content, as well as the genes *HlBx1*–*HlBx5*, the orthologs of *Bx1*–*Bx5*. The accessions of *H. vulgare* and *H. spontaneum* do not contain DIBOA [55].

In the experiments of X. Ni and S.S. Quisenberry with the accessions protected by genes of resistance to the Russian wheat aphid (*Dn1*, *Dn2*, and *Dn5*) and with the corresponding almost isogenic lines created on the basis of “Betta” cultivar, only the lines with the *Dn5* gene had a higher concentration of DIMBOA [56]. At the same time, *Dn5* and the genes that control DIMBOA biosynthesis are localized in different chromosomes. In many cases, no explicit relationship was observed between the DIMBOA content in maize plants and the degree of damage by aphids, which indicates the existence of several resistance mechanisms. It is supposed that the resistance of maize and wheat is due to the combined action of DIMBOA and other compounds such as aconitic acid [57,58].

The aphicidal and deterrent effect of indole alkaloids in cereals, of gramine first of all, on the greenbug has been shown in [59,60,61]. Interestingly, the biosynthesis of DIBOA excludes the synthesis of gramine in plants [55]. The genetic control of gramine biosynthesis has not been well understood yet. Increased content of gramine is characteristic of *H. vulgare* subsp. *spontaneum* and a number of *H. vulgare* cultivars. The presence of gramine in adult plants of *H. vulgare* subsp. *spontaneum* is controlled by one or two genes, while the content of gramine can also be controlled by minor genes [62]. At the same time, an analysis of 150 doubled haploid barley lines obtained from the crossing of “Steptoe” (high content of gramine) and “Morex” (traces of gramine) showed that the *grm* gene, which controls the synthesis of gramine in the seedling phase, is localized on chromosome 5 and is not linked to minor genes of resistance to grain aphids localized on chromosomes 2 and 5 [63,64]. An analysis of another series of doubled haploid lines also showed that QTLs, which control resistance to *R. padi* and the content of gramine in plants, are not linked [65].

To elucidate the differential mechanisms of plant defense against aphids, A. Singh et al. [66] performed a study of the aphid feeding behavior on 203 accessions of wild emmer wheat *T. turgidum* ssp. *dicoccoides* (Korn.) Thell. differing in anatomical traits (trichome density) and metabolite (BXDs) compositions. The trichomes and DIMBOA abundance were shown to be the main factors that define the efficiency of defense strategy, with trichomes being more effective compared to BXDs. The trichome density and BXDs levels depend on the genetic background in wild emmer wheats that indicates a possibility of using wild emmer wheat accessions rich in trichomes and BXDs in breeding for improved resistance to aphid.

Some key genes/mechanisms involved in providing aphid resistance/susceptibility were revealed in non-cereal crops. For example, the *PHYTOALEXIN DEFICIENT 4* (*PAD4*) gene functions as a key player in modulating defense in *Arabidopsis* against green peach aphid *Myzus persicae* (Sulzer), an important pest of a wide variety of plants [67]. The molecular mechanisms of resistance against bird cherry-oat aphids can be associated with the tryptophan-derived compounds, tryptamine, and serotonin, which accumulate in tissues of *Setaria viridis* (L.) P.Beauv. (green foxtail) plants [68].

It should be pointed out that the substances of secondary metabolism are usually concentrated in storage tissues. Therefore, the aphids, which penetrate the phloem mainly intercellularly, are able to avoid the harmful effects of these compounds. In this regard, an important role may be played by the structure of pectin, a biopolymer that functions in plants as an intercellular cement and affects the ability of aphid stylets to penetrate into the phloem. This is how an increase in the content of methoxyl groups in pectin increases greenbug resistance. Insects with increased activity of pectin methylesterase and polysacharase can feed on some previously resistant varieties of sorghum [69]. The polysaccharide matrix plays an important role in the development of relationships between aphids and host plants: most polysaccharides inhibit the feeding of the greenbug [70].

## 4. Aphid–Host Plant Interaction

The acceptability of plants to feed on determines microevolutionary processes in insect populations. The inherent heterogeneity of aphids (alternation of amphimixis and parthenogenesis) yields a combination of advantages of the two types of reproduction. During the reproduction of parthenogenetic generations, a rapid increase in aphid populations occurs when each individual reproduces its own kind, which favors the preservation of any variation of the karyotype in the populations, as all mutations persist. The autumn amphigonic generation allows aphids to survive by producing the wintering eggs and serves as a source of genetic variation. The genetic diversity in aphid populations is maintained by gene and chromosomal mutations, recombination, and assimilation of immigrants; heterogeneity of populations provides material for natural selection. These adaptive mechanisms have led to the spread of aphids throughout the world, with the greatest abundance in the temperate climate [18]. Genetic adaptation of phytophages to the plants they feed on is a widespread and well-documented phenomenon. Intraspecific forms of aphids (biotypes) that differentially interact with the host plant genotypes differ in virulence, i.e., in the ability to overcome the host’s resistance.

Genetic mechanisms of host–parasite relationships, as well as their co-evolution, were made clear by H. Flor, who studied the genetics of resistance of flax to rust and of virulence of this disease agent, *Melampsora lini* (Ehrenb.) Lév. According to the H. Flor’s “gene-for-gene” postulate [71], each resistance gene of the host has a corresponding specific virulence gene of the parasite. Mutation of virulence in the parasite determines the loss of efficiency of the host’s resistance gene. Resistance is observed when the dominant (functional) allele of the resistance gene interacts with the dominant allele of the virulence gene. Susceptibility is observed if the interacting alleles of one or both partners are found in the homozygous recessive state. An important consequence of the H. Flor’s postulate is the possibility of determining the host plant genotype without hybridological analysis by using pathogen and pest isolates marked with a certain virulence. If a parasite isolate, avirulent to the given resistance gene, damages the studied variety, this means that the variety does not have this gene.

Based on the results of studies of virulence genetics, the “gene-for-gene” relationship has been demonstrated quite substantially for a significant number of parasite–host pairs, including the systems of the greenbug–wheat and greenbug–sorghum interaction [72,73]. Experiments with three biotypes of *S. graminum* (C, E, and F) showed that virulence to the genes of wheat resistance *Gb2* or *Gb3* is determined in aphids by two recessive genes and a dominant modifier(s) epistatic to one of these genes. Nevertheless, the authors consider the observed interaction to be consistent with the “gene-for-gene” pattern when different virulence genes control the same gene product.

The same data can be explained in a way that is closer to the classical understanding of the “gene-for-gene” relationship. If one assumes that the *Gb2* and *Gb3* genes are two closely linked resistance genes in each case, then two aphid virulence genes are required to overcome them. The following data testify in favor of the linkage of genes inherited as one gene, *Gb2* or *Gb3*. The *Gb2* gene of the “Amigo” cultivar, which had been transferred from “Insave FA” rye, is known to cause antibiosis and tolerance to the biotype C, but it is ineffective to E. The initial rye cultivar has three types of resistance to both biotypes, i.e., is protected by at least two genes, one of which has not been transferred to wheat. At the same time, “Amigo” is characterized by antixenosis to the biotype E but not to C [33]. Probably, the second gene of “Amigo” with a weak effect was masked by the action of the major gene for resistance to biotype C. The accession “Largo” carrying the *Gb3* gene from *Aegilops tauschii* Coss. possesses antibiosis and tolerance to biotypes C and E. Antixenosis was also revealed in some experiments [74] but not in the others [75]. “Largo” was shown to be susceptible to biotype B; however, as in the previous case, it retained some antixenosis [76]. In addition, the “Amigo” × “Largo” F_1_ hybrid is more resistant to the biotype E as compared to “Largo” [77]. The additive effect of genes can be explained here by the presence of a minor gene for resistance to biotypes C and E in “Amigo”. The data obtained somewhat later indicated that the resistance of “Largo” and its derivatives is controlled by multiallelic complementary genes, i.e., *Gb3* should be one of the identified loci [78].

The literature data on the specific interaction of cereal aphids with host genotypes are quite numerous. For the first time, differences in the ability to feed on certain varieties of wheat and barley were found in 1947 for *S. graminum* populations in the United States [79]; however, targeted studies of the pest-intraspecific variability were not carried out until the 60s of the last century. Ten aphids biotypes identified from 1961 to 1997 were found to differentially interact with different host plants, e.g., A–C, E–K [80]. When testing aphid clones collected in four U.S. states on 16 differentiators (sorghum, wheat, barley, and rye accessions), 16 clones with 13 previously unknown virulence phenotypes were revealed [81]. In 2010, it was announced about the discovery of 13 new biotypes [82] and about six more in 2016 [83].

A long-term analysis of the Krasnodar (North Caucasus, Russia) population of *S. graminum* revealed a high polymorphism (both general and seasonal) of the insect in terms of frequencies of virulence to six sorghum accessions protected by the major resistance genes. A total of 42 virulence phenotypes (biotypes) of aphids were identified, and from 18 to 36 are being identified annually. It was found that this variability also depends on the plant resistance genes with a weak phenotypic manifestation [84,85].

Initially, some wheat accessions resistant to the Russian wheat aphid in South Africa were found to be susceptible in the United States [86]. In 2003, a pest outbreak was observed in the state of Colorado in crops of the “Prairie Red” cultivar protected by the *Dn4* resistance gene. A new intraspecific form (biotype 2, later designated RWA2) severely damages all the previously identified resistance donors, except for the accessions with the *Dn7* resistance gene [87,88]. In 2003, three new aphid biotypes (RWA3–RWA5) were identified, one of which severely damaged the accessions carrying the *Dn1*–*Dn9* genes [89], and later, RWA6–RWA8 biotypes were identified [90].

The data on the specificity of relationships between host plants and other cereal aphids, namely the corn leaf aphid [91] and the English grain aphid [92,93], are scanty.

## 5. Mechanisms of Active (Induced) Resistance of Cereals to Aphids

Currently, an increasing number of works is devoted to the induced (active, according to N.I. Vavilov) resistance of plants to phytophages, which link the mechanisms of resistance to hypersensitivity—a protective reaction of a plant expressed in the rapid local death of cells in response to the penetration of a harmful organism, accompanied by the accumulation of toxic products in dead cells. Hypersensitivity is typical for plant resistance to phytopathogens and is observed when aphids occupy various agricultural crops [94]. The induced resistance of cereals to the Russian wheat aphid is being studied especially actively; the literature on the greenbug is less extensive.

The pest–plant interaction includes several stages: secretion of inducers (elicitors), recognition of elicitors by a plant cell using receptors, signal transduction into the genome, activation of the transcription of immune response genes, and synthesis of protective compounds. Plant responses to Hemiptera insects share many common features with the reactions to phytopathogens. There are two types of mechanisms involved in internal and external lines of defense. The external (basal) line of plant defense is provided by the transmembrane pattern recognition receptors (PRR) located on the cell surface, which recognize conservative pathogen-associated molecular structures (patterns) (PAMP), such as lipopolysaccharides, peptidoglycans, and bacterial proteins. The main transmembrane receptors are receptor-like kinases (RLK) and receptor-like proteins (RLP). These PRRs induce pattern-triggered immunity (PTI). The internal line of defense is associated with effector proteins delivered inside the plant cell. This is effector-triggered immunity (ETI) provided by the R-gene encoded cytoplasmic receptors, most of which belong to the conservative family of NLR proteins, characterized by the presence of nucleotide-binding site (NBS) and leucine-rich repeat (LRR) domains. Effector proteins can be recognized either directly by cell NLR receptors or indirectly through modifications of host NLR-associated proteins [95,96,97]. The NBS-LRR gene clusters were mapped on the chromosome regions carrying resistance to corn leaf aphids in barley [98]. With the use of a PCR-based approach, the ESTs (Expressed Sequence Tags) homologous to NBS-class Resistance Gene Analogs (RGA) were identified in resistant wheat genotypes infested by Russian wheat aphid [99]. So far, only four plant NBS-LRR genes have been identified, which were shown to interact with insects [100]. Two of them are involved in controlling aphid resistance in crops. In tomato *Lycopersicon peruvianum* (L.) Mill., the *Mi-1* gene belongs to the CC-NBS-LRR (coiled coil–nucleotide-binding site–leucine-rich repeat) subfamily. It confers a dual resistance both to the root-knot nematode *Meloidogyne incognita* Kofoid and White and certain biotypes of potato aphid *Macrosiphum euphorbiae* Thomas [101]. Another NBS-LRR gene, *Vat*, confers resistance to *Aphis gossypii* Glover and some viruses in melon [102].

In barley, an active protective role can be played by phytophage hydrolase inhibitors, which are present mainly in the storage organs of plants and the accumulation of which is induced by the damage done by insects. For instance, the content of trypsin and chymotrypsin inhibitors in leaves was increasing during the colonization of barley by *S. graminum* and *R. padi*, and the accumulation was the most intense in the aphid-resistant cultivar “Frontera” [103]. The infestation of barley by *D. noxia*, *S. graminum*, and *R. padi* is accompanied by the release of ethylene [104,105]. The feeding of *S. graminum* on the resistant cultivar “Frontera” led to a rapid accumulation of hydrogen peroxide and an increase in peroxidase activity [105].

It has been shown that the colonization of sorghum by *S. graminum* and of wheat by *D. noxia* induces the accumulation of phenols and pathogenesis-related (PR) proteins [106,107,108]. So, insects induce the accumulation of chitinases, β-1,3-glucanases, and other compounds in resistant varieties; the former play an important role in the processes leading to the appearance of a hypersensitive reaction in the plant tissue. Apparently, the main elicitors of resistance, in this case, are glycoproteins [108,109,110,111]. The recognition of the feeding aphids by a plant leads to the activation of signaling systems, and there occurs a manifold increase in the concentration of such compounds as jasmonic and salicylic acids, ethylene, etc. [94].

It has been shown, for example, that the Russian wheat aphid is recognized by plants with the help of the NADP-oxidase signaling system. The feeding of *D. noxia* on the resistant cultivar “Tugela DN” led to a rapid accumulation of hydrogen peroxide and salicylic acid, as well as to an increased peroxidase activity [112,113]. A study of differential gene expression during the feeding of the Russian wheat aphid on wheat with the *Dnx* resistance gene made it possible to identify sequences similar to *Pto* and *Pti*—the genes that are involved in the tomato–bacteria *Pseudomonas savastanoi* (Janse) Gardan interaction according to the “gene-for-gene” rule [114]. The plants inhabited by the Russian wheat aphid with an expressed *Dnx* gene were found to contain over 180 genes associated with signaling and protective functions. In addition, it has been shown that the lipoxygenase signaling system may play an important role in phytophage recognition [115]. The infestation of plants carrying the *Dn7* resistance gene by two *D. noxia* biotypes led to the activation of several signaling systems, that is, Ca^2+^-phosphoinositide, lipoxygenase, and NADPH-oxidase. The plants on which aphids of the RWA1 biotype were feeding have demonstrated the differential expression of a larger number of genes compared with the plants inhabited by RWA2 (a biotype with a wider spectrum of virulence) [116,117]. A study of gene expression during the infestation by *D. noxia* of almost isogenic wheat lines characterized by different types of resistance—Tugela-*Dn1* (antibiosis), Tugela-*Dn2* (tolerance), and Tugela-*Dn5* (antixenosis and weak antibiosis)—made it also possible to reveal the difference between the signaling systems activated in plants [118].

By using almost isogenic wheat lines (a susceptible one and another with the *Gb3* gene for resistance to *S. graminum*), Y. Weng et al. demonstrated the manifestation of systemic resistance in plants induced by insect feeding [119]. The transcriptomic studies have demonstrated that sorghum plants coordinately regulate defense gene expression after an attack by *S. graminum*; however, the aphids were able to avoid triggering activation of some otherwise potentially effective plant defensive pathways, possibly through their particular mode of feeding [120].

Initiation of physical and chemical response was shown to start soon after the onset of aphid feeding, and the production of specific metabolites can have a major effect on aphid–plant interaction. So, the analysis of gene expression and metabolic dynamics in maze leaves infested by corn leaf aphids has revealed the dramatical transcriptional and metabolomic changes during the first few hours after initiation of feeding. Aphid performance was increased on the transposon insertion mutants of three BXDs biosynthesis genes, *Bx1*, *Bx2*, and *Bx6* and greatly decreased by transposon insertion in a homolog of the terpene synthases TPS2 and TPS3 [121].

In a number of recently published review articles, the mechanisms both of constitutional and induced resistance to aphids are comprehensively considered. A schematic representation of immune response involving PTI induced by plasma membrane pattern recognition receptors (PRRs) and ETI (effector-triggered immunity) in plant–aphid interactions was drawn in [122]. The schemes illustrating aphid–plant interaction during feeding and signal networks associated with plant defense are presented in [123,124]. A model of recognition of aphid feeding by resistant and susceptible plants and a scheme of plant signaling pathways involved in aphid resistance and aphid defense response signaling are presented in [94]. The main steps in the activation of plant defense responses to herbivore-associated molecular patterns resulting in compatible plant–arthropod interactions (plant susceptibility) or incompatible interactions (plant resistance) are summarized by C.M. Smith and S.L. Clement [125].

## 6. Genes of Aphid Resistance in Cereal Crops

There are three types of genetic control of resistance: oligogenic, polygenic, and cytoplasmic. The most studied is the genetics of resistance of cereal crops to the greenbug and Russian wheat aphid. The overwhelming majority of works reveal the specific oligogenic resistance of plants to pests; only sorghum is the case for discussing cytoplasmic resistance to *S. graminum* [126,127].

The allelism of resistance genes is difficult to distinguish from the tight linkage. In both cases, the contrastingly different damage is observed as a result of interaction with different biotypes of the pest, and the absence (in the case of allelism) or very rare occurrence (in the case of linkage of nonresistant phenotypes) is observed in F_2_ hybrids from crosses of resistant forms.

The non-allelic interactions of resistance genes are known, that is, epistasis, complementation, and the additive effect. Epistasis is manifested in the way that genes with low expressivity are not manifested in the presence of highly expressive genes. Their manifestation is masked by high resistance, which depends on the major genes. The genes with low expressivity are usually manifested when the major genes lose effectiveness. Complementation, in its essence, may not be different from the additive effect. If the degree of the resistance gene expression is below the phenotypic manifestation threshold, then it can manifest itself in the presence of the second gene, which also does not have an individual phenotypic expression. The interaction, in this case, resembles complementation, though, in fact, it is a manifestation of the additive effect of resistance genes.

The realizing plant genotype depends on the biotype of the insect, i.e., different resistance genes in one and the same cultivar can be expressed against different phytophage populations. As a rule, resistance genes that appear in the sprouting phase (the juvenile genes) act throughout the life of plants. At the same time, the expressiveness of resistance can change during plant ontogenesis. Resistance genes can differ in the stability of manifestation, which depends on the external environment and genetic background. For example, the sorghum hybrid “Cargill 607E” loses its resistance to the greenbug biotype I at low temperatures [128]. It was shown that the GRS 1201 and GRS 1204 wheat lines are protected by *Gb6*, a gene of resistance to *S. graminum*. At the same time, the level of expression of resistance in the GRS 1204 line is lower, which is associated with the difference in the genetic background [129]. The expression of the *Dn1* gene for wheat resistance against Russian wheat aphids can also be influenced by their genetic background [130].

### 6.1. Genes Controlling Aphid Resistance in Wheat

A systematic study of the inheritance of wheat resistance to the greenbug has been carried out in the United States since the late 1950s. Studies of a vast gene pool have identified a very small supply of resistance genes. So, to date, 15 *Gb* genes of phytophage resistance have been identified in wheat (Table 1). Among them, only 2 alleles belong to *Triticum aestivum* L., 10 have been transferred from *Aegilops tauschii* Coss., 1 from *Ae. speltoides* Tausch, and 2 from *Secale cereal* L. Most genes, with the exception of *Gb1*, are dominant and are expressed throughout plant ontogenesis. All the genes mentioned in Table 1 provide high resistance against individual insect biotypes. Only one resistance gene, *Gb3*, has been widely used in breeding programs in the southern Great Plains [131].

A number of identified resistance genes have temporary symbols. The *Gby* gene identified in the Sando’s selection 4040 line is localized on the 7A chromosome [137]. Tolerance to the greenbug biotype I displayed by the bread wheat line KSU97-85-3, which has *Ae. tauschii* 1675 in its pedigree is controlled by the dominant gene *Gbz*, which is localized on the long arm of the 7D chromosome and is allelic or closely linked to the *Gb3* resistance gene [144]. Five more dominant genes were identified in line with the genetic material of *Ae. tauschii*: the *Gbx1* gene in the accession KS89WGRC4 (Wichita/TA1695//2*Wichita), *Gba* in TA4152L94 (CETA/*Ae. tauschii*), *Gbb* in TA4152L24 (CROC 1/*Ae. tauschii*), *Gbc* in TA4063.1(68111/RUBGY//WARD/[TA2477]), and *Gbd* in TA4064.2 (ALTAR 84/[2481]). Similar to the *Gbz* gene, *Gbx1*, *Gba*, *Gbb*, *Gbc*, and *Gbd* are localized on the long arm of the 7D chromosome. It is assumed that the *Gbd* gene is different from *Gbx1* or *Gbz*. The genes *Gbx1*, *Gba*, *Gbb*, *Gbc*, and *Gbd* are either allelic or closely linked to the *Gb3* resistance gene [141].

QTLs associated with weak expression of resistance have been identified. For instance, the use of doubled haploid substitution lines in the synthetic hexaploid synthetic 7D (*T. dicoccoides* (Körn. ex Asch. and Graebn.) Schweinf. × *Ae. squarrosa* L.) (AABB × DD) has shown that the 7D chromosome contains two QTLs that cause antibiosis to *S. graminum*, and two more causing antibiosis to *D. noxia*, as well as two QTLs controlling antixenosis to the Russian wheat aphid [162]. By using a similar approach, a QTL of antixenosis to the greenbug was identified in the substitution line Chinese Spring (synthetic 6A) (*T. dicoccoides* × *Ae. tauschii*) on the 6A chromosome near the centromere, and the second QTL controlling antixenosis to *D. noxia* was found on the long arm of the chromosome 6A. This is the first time that the localization of resistance genes to two aphid species on the 6A chromosome was reported [163].

Interesting work linking resistance genes and immune response was carried out using a series of substitution lines created with the *S. graminum*-susceptible cultivar “Chinese Spring” and the resistant synthetic *T. dicoccum* × *Ae. tauschii*. The biomass, as well as the content of carbohydrates and soluble proteins, were compared in plants of these lines and parental forms infested by *S. graminum*, as well as in the control, non-infested ones. The biomass of plants of the substitution lines 5A and 6A was similar in the two variants of the test. Previously it was demonstrated that these lines show antixenosis against the insect and apparently carry genes conferring constitutional resistance. The substitution lines 1A, 1B, 7B, and 7D infestation by aphids led to a significant increase in the content of proteins. In previous experiments, the lines were characterized by antibiosis against aphids, i.e., antibiotic resistance may be associated with the expression of genes responsible for protein synthesis. The highest content of carbohydrates during colonization by insects was found in the lines 1D and 6D, which carry genes of tolerance to *S. graminum*. It is supposed that an increase in carbohydrate content causes more intensive plant growth [164].

Oligogenic inheritance of resistance is also revealed when studying the interaction of grain crops with the Russian wheat aphid. This species has become a major cereal pest in the United States and South Africa in a short period of time. Most often, resistance is found in the forms originating from Central Asia and the Caspian Sea area, i.e., the regions where the pest is endemic [165]. During approximately 25 years of intensive research, 10 wheat resistance genes have been identified and assigned permanent symbols. Most of the genes are located on the 7D chromosome. Only the *Dn4* gene, identified in the cultivar “Turtsikum 57” from the USSR and widely used in the breeding of commercial varieties, however, lost its effectiveness (such as a number of other genes) in 2003 with the appearance of a new aphid biotype RWA2. The highest level of resistance to the Russian wheat aphid biotypes currently prevailing in wheat crops is provided by the *Dn10* gene; the efficiency of *Dn2401*, *Dn2414*, Dn7, and *Dn626580* is somewhat lower [166].

The *Dn* genes localized on the 7D chromosome are characterized by cluster organization. It was shown that in the bread wheat accession, PI 294,994 from Bulgaria resistance is controlled by the dominant *Dn5* gene located on the long arm of chromosome 7D [153]. At the same time, there are data on the control of a trait by two—a dominant and a recessive [167], or two dominant genes [149]. Y. Zhang et al. showed that the contradiction in the obtained data is due to the heterogeneity of the accession PI 294,994 [168]. Segregation in F_2_ from a cross of the lines isolated from PI 294,994 with a susceptible tester indicated monogenic or digenic control of the trait. It was also supposed that PI 294,994 carried three resistance genes: two on the long arm of the 7D chromosome and one on the short arm of the chromosome 1D. X.M. Liu et al. showed that the *Dn1*, *Dn2*, and *Dn5* genes are closely linked and localized not on the long but on the short arm of the 7D chromosome near the centromere [157]. The microsatellite marker *Xgwm111* located on the 7DS chromosome is closely linked to *Dn1*, *Dn2*, *Dn5*, and *Dnx*. A resistance gene designated by the symbol *Dn8* was localized on the short arm of chromosome 7D, and the *Dn9* gene on the long arm of chromosome 1D in the accession PI 294994, i.e., as previously assumed, PI 294,994 is protected by three resistance genes. The *Dnx* gene identified in the accession PI 220,127 from Afghanistan is presumably new but may be allelic to the *Dn6* gene. A subsequent revision showed that the *Dn5* gene is localized on the long, instead of the short, arm of the 7D chromosome [154].

The localization and relationships of the *Dn1*, *Dn2*, *Dn4*, *Dn5*, *Dn6*, and *Dnx* genes, as well as the genes for resistance to the Russian wheat aphid, have been studied in a number of wheat accessions. The resistance genes in four accessions (PI 47545, PI 222666, PI 222668, and PI 225245), as well as *Dn1*, *Dn2*, *Dn5*, *Dn6*, and *Dnx*, are closely linked to the microsatellite markers *Xgwm44* and *Xgwm111* localized on the short arm of the chromosome 7D. When testing allelic relationships, segregation for resistance was not revealed in F_2_ from crosses of resistant forms with each other. Therefore, the above-mentioned genes are either alleles of the same locus or are closely linked members of the *Dn* resistance genes family. The *Dn4* gene and the previously uncharacterized *Dn* gene from the accession PI 151,918 are either allelic or closely linked and localized on the short arm of the 7D chromosome [169].

The use of microsatellite markers proposed by X.M. Liu et al. [169] has shown the presence of the *Dn4* gene in seven local wheat accessions from Pakistan, Iran, and Uzbekistan; 3 accessions from Pakistan and Tajikistan have a block of *Dn1*, *Dn2*, *Dn5*, *Dn6*, and *Dnx* genes [170], i.e., these resistance genes are found in a very diverse material from Asian countries.

The Russian wheat aphid causes not only yellowing of plant tissue by the degree to which phenotypes in hybrid populations are usually classified but also leaf rolling. The non-rolling of leaves in resistant lines W-162 and W-134 is under the digenic control. Less chlorotic F_2_ plants usually had non-rolled leaves, but also there were other variants, e.g., green (i.e., resistant) rolled and yellow (susceptible) straight leaves [171]. J.H. Peng et al. [172] identified 28 SSR loci associated with leaf chlorosis and 8 more with rolling. New chromosomal regions associated with resistance to the RWA2 biotype of the Russian wheat aphid, and the presence of new *D. noxia* resistance genes localized in homeologous groups other than bread wheat groups 1 and 7, were also identified.

By using a series of doubled haploid lines obtained from crosses of winter wheat varieties “Spark” and “Rialto”, QTLs controlling tolerance to a *D. noxia* population from Argentina were identified on several chromosomes: on 4DS (two genes) and on 5DS, 3BS, 3AS, and 7AL. In addition, antibiosis QTLs were identified on the chromosomes 4A, 1B, and 5B. It was proposed to designate the new genes as *QDn.unlp* genes [173].

Inheritance of resistance to such widespread species as the English grain aphid and the bird cherry-oat aphid has been poorly studied. The durum wheat accession C273 carries the dominant *Sa1* gene of resistance to *S. avenae* localized on the long arm of the 6A chromosome [161]. Bread wheat lines Linyuan 207, J231, and J248 each have one effective dominant gene of resistance to the English grain aphid [174,175].

Synthetic hexaploid wheat CWI76364 (*T. dicoccum* PI 94623/*Ae. tauschii* WX1027) carries the bird cherry-oat aphid antibiosis QTL on the long arm of the 4B chromosome, as well as two tolerance QTLs on chromosomes 5AL and 5BL [176]. It was shown that the spring bread wheat variety “Delfi 400” (Kazakhstan) has two dominant complementary genes that control *R. padi* antixenosis and antibiosis [29].

We are aware of only one publication on the inheritance of wheat resistance to yellow sugarcane aphid *Sipha flava* (Forbes). O.G. Merkle and K.J. Starks found that the resistance of wheat to this pest had been transferred from *Ae. tauschii* and is controlled by a dominant gene. Recently, there have been increasing reports of the growing harmfulness of the hedgehog grain aphid, a new species for the American continent [177]. The use of a series of doubled haploid lines from “Spark” and “Rialto” has revealed QTLs controlling tolerance to a population of *S. maydis* from Argentina on the chromosomes 1A, 1B, 2A, and 2D [178].

### 6.2. Genes Controlling Aphid Resistance in Barley, Oats, and Rye

To date, only four alleles of greenbug resistance are known in barley. Back in 1945, I.M. Atkins, R.G. Dahms identified two Korean winter barley varieties, “Omugi” and “Dobaku”, showing high heritability of the resistance trait [179]. Quite a few commercial varieties have been produced using “Omugi”. An analysis of the inheritance of aphid resistance showed that “Omugi”, “Dobaku”, “Derbent”, “Kearney”, and “Will” have a common dominant resistance gene primarily designated *Rsg1a* and later re-designated *Rsg1* [180,181,182,183]. The trisomic analysis has shown that the resistance gene of the “Will” variety is localized in the centromeric segment of the chromosome 1 [183], and the use of molecular markers allowed mapping of the *Rsg1* locus on the long arm of the chromosome 3H [184]. The variety “Post” [185] was created by individual selection from a hybrid population of “Harrison” × “Will”, and the heterogeneity of this variety in terms of aphid resistance necessitated the selection of the variety “Post 90” [186]. The *Rsg1a* gene controls resistance to aphid biotypes B–G, I–K, CWR, and WWG, but not to H [187,188,189,190].

The second dominant gene, *Rsg2b*, which confers resistance to the same aphid biotypes as *Rsg1a*, was identified in a local accession from Pakistan PI 426,756 [187,190,191]. At the same time, the expression of the *Rsg2b* gene is somewhat lower than that of *Rsg1a*, i.e., it is better to use the variety “Post 90” in breeding [192]. However, in subsequent experiments [193], PI 426,756 was more resistant to the E biotype compared to “Post 90”. Moreover, the *Rsg2b* gene, unlike *Rsg1a*, was effective against the aphid TX1 isolate, i.e., the differential insect–host plant interaction was observed. Based on this, new gene symbols, *Rsg1* and *Rsg2*, have been proposed. The variety “Wintermalt” is little damaged by biotypes G and J, while it is susceptible to all other intraspecific forms of the insect [188,190]. In addition to “Wintermalt”, biotype G resistance is also possessed by varieties “Colter” and “Bancroft”, which are recommended for use in breeding [194]. Subsequently, “Wintermalt” and “Colter” were shown to be severely damaged by the TX1 aphid biotype [193].

The complexity of the *Rsg1* locus has now been shown: an accession of *H. vulgare* ssp. *spontaneum* WBDC336 (PI 682028) has the *Rsg1.a3* allele, which ensures resistance to such greenbug biotypes as C, E, H, I, WY81, WY12 MC, and WY86 [195]. It has also been shown that *H. vulgare* ssp. *spontaneum* accession WBDC053 (PI 681777) carries the *Rsg2.a3* allele, which is closely linked to the *Rsg2* locus or is an allelic variant of *Rsg2*. Accession WBDC053 is resistant to the biotypes B, C, E, I, TX1, WY4A, WY4B, WY81, WY12MC, and WY86; however, it is severely damaged by aphid biotypes F, H, WY10MC, and WY10B [196].

Large-scale studies related to the search for and creation of donors of barley resistance to *D. noxia* have been carried out in the United States. As a result of the evaluation of 24,800 accessions from the USDA-ARS National Small Grains Collection, 39 forms (mainly from Afghanistan and Iran) were found to be highly resistant to the Russian wheat aphid, and 181 were moderately resistant [197].

Two lines, STARS-9301B (PI 573080, a selection from the Afghani accession PI 366450) and STARS-9577B (PI 591,617 selected from the accession Ci*ho* 4165 collected by N.I. Vavilov in Afghanistan), were soon recommended for the use in breeding [198,199]. The STARS-9301B line was found to contain an incompletely dominant *Rdn1* resistance gene (initially designated *Dnb1*) and a dominant *Rdn2* (=*Dnb2*) resistance gene. Recessive epistasis of *Rdn2* on *Rdn1* was revealed [200]. Barley lines PI 366,444 and PI 366,453 from Afghanistan have two either common or closely linked aphid resistance genes. Linkage of one of the resistance genes to the B-hordein STS marker located on the short arm of chromosome 5 was shown [201]. Resistance genes in these accessions from Afghanistan and STARS-9301B are believed to be identical [200]. In the STARS-9301B line, the *Rdn1* and *Rdn2* resistance genes were localized on the short arm of the 1H chromosome and on the long arm of the 3H chromosome, and also the third gene, *Rdn3*, was localized on the 2H chromosome [202]; the STARS-9577B line carries two resistance genes, *Rdn1* and *Rdn2* [203].

The resistance revealed in the STARS-9301B accession at the germination stage is clearly manifested in adult plants as well. By now, spring commercial varieties “Burton” (resistance donor STARS 9301B) and “RWA 1758” with resistance from STARS 9577B [204,205] have been obtained, which display effective resistance against five aphid biotypes in the USA [206]. Not only these two donors are involved in the breeding, but also other accessions with resistance to the Russian wheat aphid. For instance, by using seven barley accessions from Iran and Afghanistan, winter backcross lines were selected on the basis of the “Schuyler” variety [207]. Subsequently, in 2007 alone, 43 spring lines with 36 different sources of resistance in the pedigree were offered to breeders [208,209], and a year later, another 7 lines were offered [210].

In Mexico, the artificial infestation of plants in a field with *D. noxia* resulted in the identification of 15 resistant spring forms [211]. The laboratory evaluation of the best accessions, ASE/2CM//B76BB and Gloria/Come, revealed antibiosis, tolerance [212], and antixenosis [213] to the phytophage. Both lines are protected by a common dominant gene of resistance to the Russian wheat aphid. No reciprocal differences have been revealed, which means that the trait is determined only from the side of the nucleus [214].

When assessing the damage caused by *D. noxia* in 76 forms of barley from Iran, 17 accessions were isolated, and the genetic control of the trait was studied under laboratory conditions in the two most resistant ones. The accession Schz.B-108 has a dominant aphid resistance gene, and the little damage to Shz.B-106 is determined by an incompletely dominant gene [215]. The resistance to *D. noxia* displayed by intergeneric hybrids of barley and *Elymus trachycaulus* (Link) Gould ex Shinners was shown to be dominant [216].

J. Weibull made a comparison of the mass of *R. padi* individuals feeding on hybrids from crossing two lines of *H. vulgare* subsp. *spontaneum* with the “Golf” variety [217]. The F_2_ populations obtained from different F_1_ plants differed in resistance and, in some cases, were more susceptible than the “Golf” variety. The obtained data are interpreted in favor of the presence of several resistance genes with the additive effect.

The Japanese scientists used doubled haploid barley lines to map the QTLs controlling aphid resistance in the TR306 line. The observation of plant colonization in the field by insects for two years has shown the prevalence of *R. maidis* and *R. padi*, as well as individual colonies of *S. graminum* and *Sitobion akebiae* Shinji. The short arm of chromosome 1 was found to contain a QTL with a strong effect; this QTL is linked to another one controlling the heading date. A minor QTL is located on chromosome 5 [218].

The genetic control of resistance to *R. maidis* was investigated in five lines of barley. The lines EB921, DL529, and K144 each have one dominant resistance gene; the monogenic recessive control of the studied trait was found in Manjula and EB2507 [219].

Information on the resistance of oats to *S. graminum* is very scanty. J.H. Gardenhire [220] showed that resistance of the accession Russian 77 (CI 2898) to the greenbug biotype A is controlled by a dominant gene, subsequently designated *Tg1*. Later, R.L. Wilson et al. identified four resistant accessions: CI 1579 (South Africa), CI 1580 (Scotland), CI 4888 (Italy), and PI 186,270 (Argentina) [221]. A study of the inheritance of resistance of three accessions to two biotypes of *S. graminum* showed that PI 186,270 and CI 1580 each have one dominant gene (*Grb1* and *Grb2*, respectively), which control resistance to biotype C; the line CI 4888 was found to contain *Grb3*, a dominant gene of resistance to the aphid biotype B. All three accessions were shown to possibly have resistance genes with a weak phenotypic manifestation to both aphid biotypes [222]. The resistance gene *Grb2* works against biotypes E [74], I [189], and only partially against F–H [188,223].

We are aware of only several publications discussing the interaction of *S. graminum* and rye. The resistance of the “Caribou” variety to biotype B is apparently determined by one dominant gene [224], which is ineffective against the aphid biotype C [225]. An Argentinian rye accession, “Insave F.A.”, carries *Rpv*, a dominant gene of greenbug resistance [226]. As a result of crossing the bread wheat variety “Chinese Spring” with “Insave F.A.”, and with rye cultivars “Elbon” and “Balbo”, an octoploid triticale cultivar “Gaucho” showing resistance to the insect biotype C was created [227]. This cultivar is also protected by a dominant gene [228]. Subsequently, the “Amigo” wheat variety was developed with resistance transferred from “Gaucho” [133]. In “Amigo”, resistance is controlled by the dominant *Gb2* gene localized on chromosome 1A (translocation 1AL.1RS) [134] and closely linked to the *Sec-1* locus [229]. In contrast to the original rye variety, “Amigo” and “Gaucho” are severely damaged by aphids of biotype E, i.e., “Insave F.A.” carries at least two aphid resistance genes [74,77]. The resistance genes of this accession are effective against biotypes B, C, E, G, H, and I [77,188,189,225] but are ineffective against biotype F [188,230]. Rye accessions CI 187 and PI 240,675 are resistant to biotype F, i.e., their resistance genes are not identical to those of “Insave F.A.” [226].

### 6.3. Genes Controlling Aphid Resistance in Maize and Sorghum

There are two known genes for maize resistance to the corn leaf aphid. The resistance of the inbred maize line Hi38-71 to *R. maidis* is controlled by a recessive *aph* gene; the line Hi34 was found to contain a recessive *aph2* gene on the short arm of the chromosome 2 [231,232].

The resistance to biotype C of *S. graminum* discovered in the accessions of wild sorghum *Sorghum virgatum* (Hack.) Stapf PI 38,108 and T.S. 1636 is controlled by two dominant complementary genes. The resistance genes in *S. virgatum* and Sudan-grain (an *S. virgatum* derivative) are identical. The use of *S. virgatum* resulted in producing highly resistant forms SA 7536-1 (Shallu Grain) and KS-30, which later were widely used in breeding [233,234]. According to D.E. Weibel et al. [235], accessions IS 809, PI 264,453 (*S. bicolor* (L.) Moench), and SA 7536-1 have monogenic, incompletely dominant resistance control. The level of resistance in IS 809 exceeded that of other accessions, which may be due to the action of minor genes. Derivatives of *S. virgatum* and the line IS 809 have lost resistance to biotype E, but KS-30 is resistant to biotypes F–H. The accessions PI 264,453 and “Capbam” are resistant to biotype E, i.e., their resistance genes differ from those of *S. virgatum*. A broom sorghum accession “Deer” and an accession of Sudan grass “Piper” are characterized by resistance to biotype B and susceptibility to C, which also evidences a difference in genetic control in these forms from those previously discussed [225].

According to A.G.O. Dixon et al. [126], resistance to biotype E in accessions PI264453, “Sarvasi”, and a number of other forms is inherited polygenically. Moreover, both accessions were found to have cytoplasmic resistance to the pest.

The grain sorghum accession KS 97 has two complementary dominant resistance genes expressed against biotype I [236].

The RFLP analysis was used to study the localization of resistance genes to four biotypes (C, E, I, K) of *S. graminum* in four sorghum accessions. The accessions BTx 623, PI 550607, Tx 2783 line (isolated from “Capbam” variety), and Tx 2737 line obtained using SA7536-1 accession were analyzed. At least nine QTLs (*Ssg1*–*Ssg9*) that affect resistance were identified in eight linkage groups. None of the loci conferred resistance to all aphid biotypes [237].

To identify QTLs of resistance of sorghum to biotypes I and K, 93 recombinant inbred lines were obtained from crossing GBIK and Redlan accessions. By using 113 molecular markers (38 SSR and 75 RAPD), 9 QTLs that control resistance and tolerance to *S. graminum* were identified. Moreover, each QTL determined 5.6–38.4% of the phenotypic variance. Four SSR markers and one RAPD marker are associated with the expression of all traits of resistance and tolerance. The authors believe that these markers are linked to genes of nonspecific resistance and tolerance, while the remaining four markers are associated with specific resistance [238].

A comparison of the genetic similarity of 26 sources of sorghum resistance to the greenbug biotype I using AFLP markers revealed a high level of polymorphism in the studied accessions, most of which were divided into two clusters [239].

The genes of sorghum greenbug resistance were identified in a number of sorghum accessions. The accession VIR-457 (PI 264453, USA) carries dominant (*Sgr1*) and recessive (*Sgr2*) resistance genes. The *Sgr1* gene was also detected in accessions i-589430 (PI 264453, Spain) and VIR-3852 (Sarvashi, Hungary). These forms are assumed to also have the *Sgr2* gene. The accessions VIR-9921 (Shallu, USA) and VIR-9922 (KS-30, USA) have an incompletely dominant *Sgr3* resistance gene. The dominant gene in the accession VIR-6694 (Deer, USA) was assigned the symbol *Sgr4*. Dominant (*Sgr5*) and recessive (*Sgr6*) genes were found in accessions VIR-1362 (Durra white, Syria) and VIR-1240 (Dzhugara white, China). The variety “Sorgogradskoe” (VIR-9436, Russia) has the *Sgr5* gene. It is assumed that the *Sgr5* and *Sgr6* genes are present in accessions VIR-10092 (“Odessky 360”, Ukraine) and VIR-5091 (Cherhata, Morocco). The accession VIR-924 (Dzhugara white, China) is protected by the dominant (*Sgr7*) and recessive (*Sgr8*) genes. The accession VIR-923 (Dzhugara white, China) has at least one of these genes. The accession VIR-930 (Dzhugara white, China) has two dominant complementary resistance genes (*Sgr9*, *Sgr10*). One of the two dominant genes in the accession VIR-1237 (Dzhugara white, China) was assigned the symbol *Sgr11* [240].

The inheritance of resistance to the Krasnodar greenbug population has been analyzed for nine forms of grain sorghum and Sudan grass. The dominant gene of the “Capbam” accession (VIR-455, USA), which manifests itself against individual clones of the insect, differs from the previously identified *Sgr1*–*Sgr11* resistance genes and is denoted by the symbol *Sgr12*. In addition to the dominant *Sgr1* gene, the variety “Sarvashi” (VIR-3852, Hungary) is also protected by a recessive gene (obviously *Sgr2*) against individual aphid clones. Grain sorghum accessions VIR-928 and VIR-929 (Dzhugara white, Western China) have two highly effective dominant resistance genes that differ from *Sgr1*–*Sgr4*, *Sgr6*, *Sgr9*, and *Sgr10* genes. The resistance genes of the VIR-929 accession also differ from the *Sgr5* gene. The accession k-928 was found to contain a third dominant resistance gene, which is expressed against individual aphid clones. The symbol *Sgr13* has been assigned to this gene. Sudan grass accessions VIR-100 and VIR-122 (Ukraine) have two dominant genes of resistance to the insect; one dominant and one recessive resistance gene were found in accessions VIR-62, VIR-99 (Ukraine), and VIR-96 (Russia). Dominant resistance genes of the variety “Odesskaya 25” (VIR-122), which manifest themselves against some clones from the natural aphid population, are designated by the symbols *Sgr14* and *Sgr15* [241].

As in the above-considered systems of interaction between *S. graminum* and host genotypes, there are numerous data in the literature on the preservation of weak resistance in sorghum accessions following the loss of the effectiveness by the main genes [74,188]. The joint inheritance of oligogenes (*Sgr1*, *Sgr4*, *Sgr5*, *Sgr6*) and weakly expressed resistance, which manifests itself when plants are colonized by virulent insect clones, have been analyzed. It was shown that resistance, in this case, does not depend on the “residual effect” of oligogenes but on the interaction of minor genes of resistance–virulence. Minor genes can be independent or weakly linked to the major resistance gene. The differential host–insect interaction in terms of minor resistance–virulence genes was observed, as well as an increase in the frequency of clones during seasonal changes in the natural population, which are maximally compatible with the “Sarvashi” variety, which was widely used in breeding, concerning both the major and minor resistance genes [242].

The dominant gene *RMES1* ensuring sorghum resistance to an invasive species of sugarcane aphid *M. sacchari* was mapped in the Chinese grain sorghum variety “HN16” on chromosome 6 [243,244]. By using the NGS technology and transcriptomic analysis, four QTLs (intervals) containing genes of resistance to *M. sacchari* were identified in line 407B on chromosome 6, and the *qtlMs.6-1* locus contains the previously identified *RMES1* gene [245].

## 7. Gene Pool and Cereal Crops Breeding for Resistance to Aphids

The increasing genetic homogeneity of cultivated crops promotes the acceleration of adaptive microevolution of harmful organisms. The results of numerous studies show that the nature of the phenotypic manifestation and inheritance cannot distinguish resistance that can potentially be overcome by insects from the insurmountable (durable) one. Both major and minor aphid resistance genes in cereal crops interact differentially with pest genotypes. Therefore, the possibility of insect adaptation is quite obvious in both cases.

The specificity of the host plant relationship, first of all, with the greenbug has been discussed in numerous publications for over 70 years. Over the years, a fairly large number of resistant forms have been identified; some of them were widely used in breeding and, unfortunately, inevitably lost their effectiveness.

It was supposed that the reason for the long-term persistence of resistance should be sought not in the mechanisms of host–parasite interaction but in the negative consequences for the phytophage of the corresponding virulence mutation. The data obtained for the system of sorghum–greenbug interaction confirmed a hypothesis about the relationship between the rare virulence of the phytophage and reduced viability. Clones from the Krasnodar population of *S. graminum* virulent to the *Sgr5* and *Sgr6* resistance genes are less fertile compared to avirulent ones and are displaced during the reproduction of model populations on a susceptible sorghum line [246].

The useful life duration of a resistance gene in case of its wide use in breeding does not exceed 10 years. The Russian wheat aphid is a key pest of cereal crops in the United States. In 1994, wheat varieties with the *Dn4* resistance gene were released into production, and already in 2003, in Colorado, a phytophage outbreak was observed in crops of the “Prairie Red” variety protected by this gene. A new infraspecific form (biotype 2, later designated *RWA2*) quickly became dominant (73–95%) in wheat and barley crops in at least four states [247]. Another example is the cultivation in the USA of sorghum hybrids resistant to biotype E of *S. graminum*. Sales of seeds of these hybrids began in 1982; by 1986, the volume of sales in Oklahoma amounted to 38% of the total [248]. In 1989 resistant hybrids occupied about half of the sorghum area in Kansas and 90% in Texas [249]. The spread of the “sorghum” biotype I was noted in 1990, while all the previously found sources of resistance turned out to be ineffective [189]. In Russia, sorghum is a less common crop, so the predominance of aphid clones virulent to the “Sarvashi” variety widely used in breeding in southern regions of the country was noted approximately 20 years after the release of varieties and hybrids with this resistance donor in the pedigree [240].

A rational breeding strategy envisages, first of all, broadening the genetic diversity of cultivated varieties. Depending on the characteristics of the crop, the specific contribution of one or another method of expansion (search for resistant forms among cultivated species, introgression, mutagenesis) can be different.

The identification of new resistance genes from cereal collections is the easiest way to replenish their stock; however, donors of new genes are usually rare. For example, among more than 23,000 sorghum accessions evaluated by T.L. Harvey et al. in the 1980s for resistance to *S. graminum*, not a single form resistant to biotype I could be identified [189]. After an unsuccessful attempt to find forms resistant to *D. noxia* among the randomly selected 5,000 accessions of local wheat, screening of databases on 17,778 accessions held by ICARDA, Australia and Russia (VIR) genebanks has been carried out. The screening took into account such parameters as (1) the origin of accessions (regions where the pest was recorded) and yielded 10,200 accessions, (2) rainfall (aphid prefers relatively dry conditions), which shortened the list to 3338 forms, and (3) temperature and altitude, leaving 1125 accessions from 521 locations on the list. The resistance of 510 genotypes from the ICARDA collection was assessed, and 12 more or less resistant forms were selected, among which six (from Pakistan, Iran, and Uzbekistan) are characterized by a high level of resistance to the Russian wheat aphid [250]. The molecular screening has shown that most of these identified accessions have either the *Dn4* gene or a block of *Dn1*, *Dn2*, *Dn5*, *Dn6*, and *Dnx* genes, and only two accessions are most likely protected by unknown resistance genes [170]. Nevertheless, the gene pool of grain crops is far from being exhausted, as is evidenced by extensive information.

High resistance to the phytophage is often found in local cultivated cereal species. N.I. Vavilov [30] believed that “… resistance is developed under the influence of natural selection only under those conditions that promote the development of infection, and, as a rule, are detected only where one or another parasite is present, against which resistance is developed through selection”. We found sorghum forms most resistant to *S. graminum* among local accessions from China but not from Africa (the primary homeland of the crop), which is associated with the long-standing insect–host plant relationship [240]. In a study of 1358 accessions of barley from the countries of East and South Asia, heterogeneous forms were identified that differ in the level of expression of greenbug resistance. The high resistance of 98 accessions is controlled by alleles that are not identical to alleles of the previously identified *Rsg1* gene. The occurrence of resistant forms is the highest among the material from the provinces of Shaanxi, Shanxi, and Henan in the Central Natural Region of China, which may be due to favorable conditions for aphids development and the duration of *S. graminum* coexistence with barley [251].

N.I. Vavilov wrote: “Ecogeographic correctnesses in the detection of resistance are relatively common, inherent in various plants which often belong to different genera and even families” [30]. In particular, 371 accessions of oats from the countries of Asia and the Far East of the Russian Federation were studied, and 95 forms heterogeneous in terms of resistance to *S. graminum* were identified. Seven homozygous resistant lines were selected, and these forms were shown to be protected by different alleles of resistance genes, which also differ from the *Grb3* gene [252].

At present, the introgression of resistance genes has become widespread. The main advantage of this method of expanding genetic diversity is the certainty that the source of a given gene has not been used in breeding yet. The important role of introgression is evidenced, for example, by the previously discussed results of studies on the inheritance of wheat resistance to *S. graminum*: out of 15 *Gb* resistance genes, only two alleles actually belong to *T. aestivum*. It is believed that due to differences in the structure of coding sequences, the introgressed genes provide a wider range of durable resistance compared to the genes of the recipient species. The wild barley ancestor *H. spontaneum* 5 (Hsp5) is characterized by partial resistance to several aphid species: *R. padi*, *S. avenae*, and *Utamphorophora humboldti* Knowlton. The comparative study of *R. padi* feeding behavior on infested plants of Hsp5 and a susceptible variety, “Concerto”, has revealed that the protection factors of wild barley are localized in mesophyll layers and phloem tissues. Aphid resistance was associated with the increased expression of the major genes involved in the signaling of the phytohormones jasmonate, abscisic acid, and ethylene, as well as of the protective peptides thionins. In addition, a reduced level of accumulation of essential amino acids in the phloem of the resistant genotype was observed [253].

At the same time, numerous literature data indicate that insects can overcome the resistance of varieties with foreign genes just as easily as resistance from closely related species.

In case of gene pool exhaustion, mutant forms created using traditional and biotechnological methods become of primary importance. The variability observed among somaclones (plants obtained in in vitro culture) is so great that the application of mutagens often does not increase its level. The most promising objects for studies of somaclonal variability may be *S. graminum* and *D. noxia*—the species that are believed to inject toxins into plants while feeding. For instance, R. Zemetra et al. evaluated the resistance of wheat calli to the Russian wheat aphid extract in vitro and identified three somaclonal variants with a higher level of resistance to the pest compared to that in the original form [254]. Selection can also be carried out after plant regeneration. New sources of resistance can also be obtained by targeted changes in gene sequences, in particular, with the application of gene and genome editing approaches using TALEN and CRISPR/CAS9 tools [255].

## 8. Conclusions and Future Outlooks

The interaction of two adjoint evolving systems is a characteristic feature of genetic control of plant resistance to diseases and pests. Involving as many varieties as possible in breeding and their rational use is needed. There are several ways of pest management, which are based on increasing plant populations diversity in space and time:

- Alternation of varieties with different resistance genes in time;
- Cultivation of mixtures of genetically different varieties or even crops aligned only for precocity;
- “Mosaics”, i.e., simultaneous cultivation of a large number of varieties with different resistance genes in the pest area;
- Breeding of multiline varieties, i.e., mechanical mixtures of phenotypically similar lines differing in resistance genes;
- Pyramiding, i.e., association of various resistance factors in one genotype.

These strategies are not alternatives to each other and can be used in any combination.

The slowing down of the process of pests’ adaptation to resistant varieties depends on the expansion of the bank of effective resistance genes, as well as on the knowledge of the evolutionary mechanisms of pest compatibility with host plants. A long-term study of aphid population variability in terms of virulence to host plant resistance genes is necessary in order to propose a reasonable distribution scheme for varieties with different resistance genes. Nevertheless, the creation of irregular, that is, unregulated in any way, variety mosaics is quite possible at the present time.

## Figures and Tables

**Table 1:** Wheat aphid resistance genes.

Chromosome	Resistance Genes of *T. aestivum*	Resistance Genes of Related Species
***S. graminum* resistance genes**		
No data available	*Gb1* [132]	-
1A	-	*Gb2* (*S. cereale*) [133,134] *Gb6* (*S. cereale*), linked to *Gb2* [135,136]
7A	*Gby* [137]	*Gb5* (*Ae. speltoides*) [138]
7D	-	*Gb3* (*Ae. tauschii*) [134,139] *Gb4* (*Ae. tauschii*), either tightly linked to or allelic with *Gb3* [140,141] *Gb7* (*Ae. tauschii*), linked to *Gb3* [142] *Gb8* (*Ae. tauschii*) [143] *Gbx1, Gba, Gbb, Gbc, Gbd, Gbz* (*Ae. tauschii*), either allelic with or tightly linked to *Gb3* [141,144]
***D. noxia* resistance genes**		
1B	-	*Dn7* (*S. cereale*) [145] *Dn2414* (*S. cereale*) [146]
7B	-	*Dn1881* (*T. durum*) [147]
D genome	-	*Dn3* (*Ae. tauschii*) [148]
1D	*Dn4* [149,150]	-
7D	*Dn1* [151,152] *Dn2* [150,151] *Dn1* and *Dn2*, probably, allelic [149] *Dn5* [153,154] *Dn6* [149,155], either allelic with or tightly linked to *Dn1*, *Dn2,* and *Dn5* [156] *Dn8* [157] *Dn9* [157] *Dn10* [158] *Dnx* [157] *Dn2401* [158] *Dn100695* [159] *Dn626580* [160]	-
***S. avenae* resistance genes**		
6A	-	*Sa1* (*T. durum*) [161]
